# Validation and clinical interpretation of the St George’s respiratory questionnaire for COPD (SGRQ-C) after adaptation to Malaysian language and culture, in patients with COPD

**DOI:** 10.1186/s12955-020-01393-1

**Published:** 2020-05-13

**Authors:** Anees ur Rehman, Mohamed Azmi Ahmad Hassali, Sabariah Noor Harun, Sameen Abbas, Jaya Muneswarao, Irfhan Ali Bin Hyder Ali, Rabia Hussain

**Affiliations:** 1 Department of Clinical Pharmacy, School of Pharmaceutical Sciences, University Sains Malaysia, Minden, 11800 Penang, Malaysia; 2 Faculty of Pharmacy, Bahauddin Zakariya University, Multan, Pakistan; 3 Department of Pharmacy, Quaid e Azam University, Islamabad, Pakistan; 4 Respiratory Department, Hospital Pulau Penang, Ministry of Health, Penang, Malaysia

**Keywords:** SGRQ-C, COPD, MCID, Psychometric validation, Quality of life

## Abstract

**Background:**

Cultural differences affect the administration and results of health status questionnaires. “Cross cultural adaptation” ensures retention of psychometric properties such as validity and reliability at an item and/or scale level.

**Objective:**

To develop a Malaysian version of St George’s respiratory COPD specific questionnaire (SGRQ-CM), to evaluate the full spectrum of psychometric properties (reliability, validity and responsiveness), to test the factor structure and to assess minimum clinically important difference for the SGRQ-CM, to be used in population of Malaysia.

**Methodology:**

SGRQ-C was translated to Bahasa Malaysia using a standard protocol. 240 COPD patients were included in the study. All patients were followed-up for six months. Construct validity, internal consistency, item convergent validity, test-retest ability, responsiveness, factor analysis and MCID of the Malaysian version of SGRQ-C to be used in population of Malaysia were evaluated.

**Results:**

The Cronbach alpha coefficient and intraclass correlation coefficients (ICC) for SGRQ-CM were reported as 0.87, and 0.88 respectively. Correlation of SGRQ-CM with CAT, EQ-5D-5 L, mMRC dyspnea scales and FEV_1_%predicted were reported as 0.86, − 0.82, 0.72 and − 0.42 respectively. Correlation coefficient between the subscales and other clinical and health status measures ranged from r = − 0.35 to r = − 0.87. The MCID was reported as 5.07 (− 2.54–12.67).

**Conclusion:**

The Malaysian version of SGRQ-C has a good psychometric property comparable to those of the original version and has a strong evidence of validity, reliability and responsiveness towards disease severity in Malaysian COPD patients. It can be recommended as a reliable quality of life measure for future research.

## Key Points

1. SGRQ-CM has a strong evidence of validity (construct and concurrent), reliability and responsiveness to disease severity in Malaysian COPD patients.
2. It can be used as a reliable QoL measure in future research, including randomized clinical trials, rehabilitation studies, and QoL studies in COPD patients.
3. It can be used in clinical practice to measure treatment efficacy for selection of optimal treatment.

## Introduction

In addition to spirometric values, Global Initiative for Chronic Obstructive Lung Disease (GOLD) 2019 updates recommend to assess impact of chronic obstructive lung disease (COPD) on the health status of patients, disease progression rate and exacerbation frequency prior to initiation of new therapy [1]. COPD is a multifactorial health problem, having substantial impact on patients’ financial status and quality of life (QoL) [2, 3]. COPD patients experience numerous pulmonary and extra pulmonary symptoms, including dyspnea, cough, sputum, fatigue, insomnia and systemic inflammation. These symptoms are responsible for impaired QoL and can be assessed through communication with the patient rather than spirometry. Disease activity markers (lung function’s parameters, sputum frequency, sputum volume and exacerbation), may not necessarily reflect the overall impact of the disease on an individual patient [4]. Incorporating health status questionnaires into the clinical investigations of COPD may aid in determining the treatment efficacy, disease severity and health status of COPD patients [4].

In general, QoL questionnaires are widely used to observe patient improvement, responsiveness to ongoing therapy, verify the lab test values, and effect of clinical interventions. They are also helpful in the selection of most appropriate therapy among a broad spectrum of therapeutic interventions. The SGRQ-C is a self-administered COPD specific questionnaire to evaluate the comparative measurement of health status based on severity of disease and can also evaluate the effectiveness of therapy after treatment [5]. It has adequate sensitivity and reliability. Once efficiently translated and validated, it provides a standard metric across cultures and populations [6–9]. It can evaluate disease symptoms, patient’s daily activity, the impact of COPD on patient’s life and total QoL score. Each item in the SGRQ-C questionnaire has a specific weight, which is combined to calculate subscale or total score [5]. The minimum clinically important difference (MCID) for the SGRQ-C is its response to therapeutic interventions and is used to evaluate clinical efficacy of the therapy in COPD patients. MCID is valid and correlates with clinical parameters of respiratory function e.g. forced expiratory volume in one second (FEV_1_) [10].

Cultural differences affect the administration and results of health status questionnaires [11]. Food and Drug Administration Authority (FDA) requires validation of patient reported health status measures in terms of linguistic and cultural adaptation to ensure content validity at the conceptual level among different populations before applying to a new or different population [12]. “Cross cultural adaptation” resolves translation and cultural adaptation issues in using a questionnaire in different ethnicity than the source of origin, and finds content similarity between source and target population [13]. It ensures retention of psychometric properties such as validity and reliability at an item and/or a scale level. Moreover, the translation of the questionnaire to native language not only improves communication between patient and healthcare professionals, but also helps in measuring the exact experience of individuals on QoL due to influence of the disease without any interference from the health professional [14].

Currently modified medical research council dyspnea scale (mMRC scale), COPD assessment test (CAT) and few generic health status questionnaires e.g. European Quality of life 5-Dimension 5-Level questionnaire (EQ-5D-5 L) are being used in clinical practice in Malaysia. CAT is a short form of SGRQ-C and can only measure respiratory disability and few activities among patients with COPD [15]. Whereas, generic questionnaires are less responsive to changes in disease status and are less effective for use in clinical trials as they do not focus on aspect, specific to a certain disease [16, 17]. There is a need for a scale which can measure in detail the four main domains of health status that include physiological functioning, symptoms, functional impairment, and QoL. Translation and validation of SGRQ-C to Malaysian language and culture can cover this gap, as it can assess the detailed symptomatic effect of disease and impaired QoL among COPD patients in clinical practice and research studies. Bahasa Malaysia is an official language of Malaysia and is spoken by approximately 85% of the Malaysian population [18]. The objective of our study was to develop a Malaysian version of SGRQ-C (SGRQ-CM), to evaluate the full spectrum of psychometric properties (reliability, validity and responsiveness) of the SGRQ-CM, to test the factor structure of SGRQ-CM and to assess MCID for the SGRQ-CM, to be used in population of Malaysia.

## Methodology

This study was part of a prospective cohort that included patients from the main public hospital of Penang, Malaysia. The study protocol was approved by the National Medical Research Register Malaysia and clinical research center of concerned hospital (Registration number: NMRR-18-1482-42,075). Sample size for validation of the SGRQ-C questionnaire was calculated as 240 (5 participants against each item in the instrument with 20% drop out rate) [19]. Written informed consent was obtained from all participants.

### Translation

The translated version of the SGRQ-C questionnaire was available from the St George’s library. To verify the translated version, a native Malay speaker with good command on English was selected and performed forward translation of SGRQ-C English version to Malaysian language.

A team comprising medical experts, academicians and the translator reviewed the both translated versions of SGRQ-C and developed a new translated version by resolving conflicts through discussion. To check the validity of the newly Malaysian translated version that it reflects the same content as original version, two independent translators translated the newly Malaysian translated version to the English. Then a committee of experts comprising health professionals, academicians and translators (forward translator and backward translators) reviewed the forward and backward translated version to come up with a final version of Malaysian translated SGRQ-C.

**Fig. 1:**
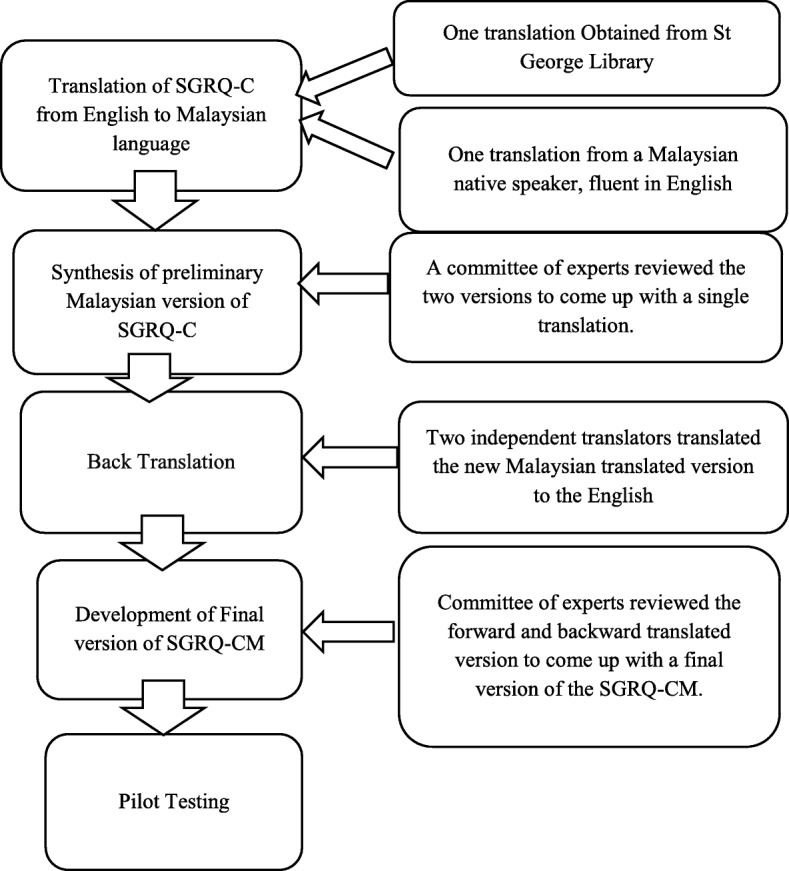
Translation and linguistic validation methodology used for translation of the SGRQ-C. *SGRQ-C, St George Respiratory Questionnaire for COPD; SGRQ-CM, Malaysian version of SGRQ-C

The translated version was reviewed critically to validate semantic, idiomatic, experiential and conceptual equivalence. To assess that the translated version still retains its equivalence to the original version, it was filled initially by 10 COPD patients and the patients were interviewed to probe what he/she thought about the meaning of each questionnaire. No difficulty was reported in understanding or answering the questions. Therefore, the Malaysian version of SGRQ-C (SGRQ-CM) was adopted as the final version of the questionnaire (Fig. 1).

### Study design

Data on clinical and health status of COPD patients was collected from the patients visiting chest clinic of the Penang Hospital. Patients were followed up for six months. Data were collected for all the included patients at baseline during recruitment, then a repeated evaluation of 20 % patients at an interval of two weeks and then follow-up evaluation of all patients at an interval of six months.

Among 240 patients included in the study forty seven patients suffered from exacerbation and admitted to the ward during the study period. For admitted patients, data were also collected on the last day of admission due to exacerbation and at an interval of one month after discharge when a significant improvement in health status was expected.

The patients were included in the study if they had, (1) confirmed diagnosis of COPD (FEV_1_/FVC < 70), (2) minimum of six-month outpatient record to avoid abrupt changes in QoL due to initiation of therapy, (3) no changes in treatment over the past 4 weeks, (4) no other respiratory disorders, (5) no other diseases that have a short term effect on QoL, and (6) no disability. Patients were clinically assessed and then administered the data collection form consisting of demographic data, clinical data, and health status questionnaires (SGRQ-CM, CAT, EQ-5D-5 L, and mMRC dyspnea scale). Help was provided by non-technical staff to complete the questionnaire, if someone was unable to complete by himself due to poor eyesight, unable to read or shaky hands. Nondirective guidance was provided if patients had queries on how to answer questions.

For SGRQ-CM questionnaire the recall period for symptom and activity subscale was “past year” and “these days” respectively. Spirometry was performed according to American Thoracic society guidelines at each visit before administration of the data collection form. Severity of COPD was categorized according to spirometry results, in accordance with GOLD 2018 guidelines [20]. Grade I COPD with FEV_1_ ≥ 80% predicted, grade II with FEV_1_ 50 to 80% predicted, grade III at FEV_1_ 30 to 50% predicted, and grade IV with FEV_1_ <  30% predicted. Stable COPD patients were defined as patients having less than 10% change in spirometric values, and no variation in clinical symptoms after two weeks.

### Health status measures

Three health status measures, CAT, the Malaysian version of EQ-5D-5 L, and mMRC dyspnea scale were simultaneously applied to the study population along with the SGRQ-CM. CAT is an FDA approved health status questionnaire used for assessment of COPD patients. CAT is easy to understand and consist of 8 items related to symptoms and activities. Each item has scores 0 to 5 from best to worst with a maximum total score of 40. Its reliability has been already tested in many European countries and literature shows the high correlation of SGRQ-C score and CAT score [21].

The EQ-5D-5 L is a generic health status measure comprising two components: a descriptive utility index (EQ-5D-5 L UI) and a visual analogue scale (EQ-VAS) [22]. The utility index is calculated from the descriptive scale of five components (mobility, self-care, usual activities, pain and depression). Patients mark each dimension on a scale of 1 “no problem” to 5 “worst problem”. The five-digit number (ranging from 11,111 to 55,555) obtained was then converted to a utility index based on EQ-5D-5 L value set for Malaysia [23]. For EQ-VAS participants were asked to mark their health on a vertical visual analogue scale from 0 (worst possible health) to 100 (best possible health). Higher EQ-5D-5 L UI and EQ-VAS values reflect good health status.

The mMRC is a self-administered FDA approved health status measure. It has been used for years to assess the level of breathlessness and its impact on daily activities on a scale from 0 to 4 (Grade 0: not troubled by breathlessness except on strenuous exercise, Grade 1: Short of breath when hurrying or walking up a slight hill, Grade 2: walks slower than contemporaries on level ground because of breathlessness, Grade 3: Stops for breath after walking about 100 m (109 yards) or after a few minutes on level ground, Grade 4: too breathless to leave the house or breathless when dressing or undressing). It is easy to use, and has a well-established relationship with spirometric values and SGRQ-C [24].

## Psychometric analyses

Construct validity (known-group validity and reliability) was assessed by analyzing the correlation of SGRQ-CM total and subscale scores with clinical measures and other health status measures (CAT, EQ-5D-5 L and mMRC) obtained during the baseline assessment. The linear trend between SGRQ-CM scores and spirometry values across different levels of disease severity was tested using Pearson’s correlation coefficient.

Internal consistency reliability of SGRQ-CM total score, symptom subscale, activity subscale and impact subscale score was assessed based on the value of Cronbach’s alpha coefficient. Internal consistency reliability for each scale is considered as excellent if Cronbach’s α is ≥0.9, strong if Cronbach’s α is ≥0.8, acceptable if Cronbach’s α is ≥0.7 and reasonable if Cronbach’s α is ≥0.6. This cut of values have been adapted from Taber, K. S. (2018) [25]. Test-retest ability was assessed using intraclass correlation coefficients (ICC) [26]. Responsiveness can be defined as the ability of the health status measure to determine clinically meaningful changes with the passage of time or under the impact of an intervention. The responsiveness of SGRQ-CM was assessed by calculating the effect size (ES). ES determines the magnitude of change and was calculated by using the mean difference and standard deviation (SD) at baseline and after six months of follow-up and between patients at different stages of disease severity. The mean difference between two groups was divided by change in SD at two time intervals. The value of ES of≥0.8, ≥0.5 and ≥ 0.2 represent strong, medium and small sensitivity respectively, and this cut of value was adapted from Cohen, J. (1988) [27].

Factor analysis evaluates the internal structure of a questionnaire. It gives the significance of each item with the hypothesized scales and the overall model fit. The three factor structure of SGRQ-C was confirmed using explanatory factor analysis (EFA) and confirmatory factor analysis (CFA). For EFA the accuracy of the model fit was confirmed with Kaser-Meie Olkan (KMO) value (good fit if KMO ≥ 0.8 and ≤ 1.00; satisfactory if KMO ≥0.7 and < 0.8; acceptable if KMO ≥0.6 and < 0.7; and unacceptable if KMO < 0.6) and a significant *p* value of Bartlet’s test of sphericity. These cut of points values have been adapted from Li, C.-H. (2016) [28]. In EFA, allocation of an item to the relevant factor was considered satisfactory if, the Item showed factor loading value of > 0.25 to the particular subscale [29]. CFA was then performed to confirm the results of EFA. To access the accuracy of model (model fit) for CFA following fit indices were used: standardized root mean square residual (SRMR), good fit if SRMR≤0.08 and acceptable if SRMR> 0.08 to < 0.1; the root mean square error of approximation (RSMEA), good fit if RSMEA ≤0.06, acceptable fit if RMSEA is > 0.06 and < 0.08; the comparative fit index (CFI), good fit if CFI ≥0.95 and acceptable fit if CFI is ≥0.90 and ≤ 0.95; the Tucker-Lewis index (TLI), good fit if TLI ≥0.90. All these values were adapted from Li, C.-H. (2016) [28]. Scaling success rate was assessed for each subscale as the percentage of items within a subscale that is highly correlated with the hypothesized subscale as compared to other subscales.

MCID indicates a clinical threshold which is used to differentiate between patients and within patients under the impact of an intervention with the passage of time and provides information about an intervention that it achieved the minimum level of perceived benefit and moves beyond the concept of statistical differences [30]. There is no single established method for measurement of MCID. Different approaches are used to assess the MCID. Anchor-based method measures MCID based on relationship between the instrument to be assessed and other measures of health status (the anchors) [31]. Using Anchor-based approach MCID was assessed by calculating the mean difference (95%CI) in baseline SGRQ-CM total and impact subscale scores among patients at adjacent disease and dyspnea level. The difference between dyspnea grade III and IV is important, as it distinguish between patients with no activity from patients with comparably better activity. Distribution-based approach involves the comparison of health status score to a clear change in patient clinical status or to a criterion of health which requires major change in treatment. The principle underlying this approach is that health status measure scores should be worst in patients underlying major health event and will improve significantly with the recovery form the event [31]. Exacerbation causes significant changes in patient’s clinical status and spirometric values and 75% recovery is expected after exacerbation in the first four weeks, which indicates a major improvement in health status [9]. Using distribution-based approach MCID was assessed by administering the questionnaire to the COPD patients being discharged from hospital after exacerbation and at one-month post-exacerbation treatment when a significant improvement in health status was expected.

It was hypothesized that SGRQ-CM would differentiate patients on the basis of their disease severity (known-group validity), has a significant correlations with clinical and health status measures (CAT, mMRC dysponea scale, EQ-5D-5 L and spirometric values) (reliability), and can detect changes in QoL under the influence of disease over time (responsiveness) [32, 33]. A *p* value less than 0.05 (two sided) was considered to be statistically significant. All analysis were performed using SPSS version 24.0 (IBM SPSS Statistics for Windows, 142 Armonk, NY: IBM Corp.) and IBM AMOS version 20.0 (Arbuckle, JL, Chicago: IBM SPSS).

## Results

A total of 240 patients who met the inclusion criteria were included in the study. Mean age was reported as 69.40 years, and mean BMI was reported as 23.49. Among the included patients 44.2% were Chinese, 24.5% were Malay, and 29.2% were Indian. 89.5% of the patients were current or ex-smoker. At baseline mean FEV1/FVC and FEV_1_% predicted were reported as 51.72 and 55.27 respectively. The mean SGRQ-CM score was reported as 49.93. The mean SGRQ-CM scores in GOLD 1, GOLD 2, GOLD 3, and GOLD 4 patients were reported as 41.89, 45.26, 53.25 and 64.30 respectively. Demographic characteristics and clinical measures of the sample population are shown in the Table 1.

**Table 1:** Demographic and clinical characteristics of COPD patients at baseline

	**Outpatient (Stable Disease)*n*** **= 240**
Age: mean Years (SD)	69.40 (6.87) kg/m^2^
BMI: mean (SD)	23.49 (4.35)
**Ethnicity: % (n)**	
Malay	24.5 (60)
Chinese	44.2 (106)
Tamil	29.2 (69)
Others	2.08 (5)
**Smoking Status: % (n)**	
Current Smoker	11.6 (28)
Ex-Smoker	77.9 (187)
Non-Smoker	10.4 (25)
Post-bronchodilator Spirometry: Mean (SD)	
FEV_1_/FVC	51.72 (12.75)
FEV1 (%)	55.27 (14.84)
**Mean (95% CI) SGRQ-CM scores at different disease stage: GOLD Classification (% Predicted FEV**_**1**_**)**	
l (> 80%)	41.89 (29.45–54.34)
ll (50–79%)	45.26 (41.32–49.19)
lll (30–49%)	53.52 (47.52–59.52)
lV (< 30%)	64.30 (56.72–71.87)
**Mean (95% CI)SGRQ-CM scores at different disease stage: mMRC Dyspnea grade**	
Grade 0	29.24 (25.9–32.58)
Grade 1	32.46 (29–35.91)
Grade 2	47.90 (42.2–53.57)
Grade 3	57.77 (53.3–62.16)
Grade 4	66.36 (60.8–71.85)
**Health status instrument Score: mean (SD)**	
CAT	16.52 (6.85)
EQ-5D-5 L UI	0.62 (0.23)
EQ-VAS	59.24 (17.30)
mMRC	2.31 (1.31)
**SGRQ-CM Scores: mean (SD)**	
Symptom	55.44 (19.98)
Activity	58.43 (18.78)
Impact	43 (20.71)
Total Score	49.93 (18.61)

*BMI* Body mass index in kg/m^2^, *COPD* Chronic Obstructive Pulmonary Disease, *CAT* COPD assessment test, *EQ-5D-5 L UI* European Quality of life 5-Dimension 5-Level questionnaire Utility index score, *EQ-VAS* European Quality of life 5-Dimension 5-Level questionnaire Visual analogue score, *FEV*_*1*_ Forced expiratory volume in 1 s, *FVC* Forced vital capacity, *GOLD* Global Initiative for Chronic Obstructive Lung Disease, *mMRC* modified Medical Research Council dyspnea scale, *SGRQ-CM* St George’s Respiratory COPD specific Questionnaire Malaysian version, *SD* Standard deviation

EFA results showed that 3-factor structure of SGRQ-CM was optimal, explaining 68.31% of the variance. In EFA, the adequacy of the factor model was confirmed, with KMO value of 0.759 and a significant *p* value of Bartlet’s test of sphericity (*p* < 0.001). Loading of SGRQ-CM item 2, 3, 6, 9.5, 12.3, 10.6, 14, and 11.5 were higher than 0.25 in more than one factors, the factor with highest loading was selected [34]. In EFA Six out of seven symptom items showed loading in symptom factor with a success rate of 87.5%. Similarly loading of relative items in Activity and impact factor showed a success rate of 84.6% (11 out of 13 items) and 80% (16 out of 20 items) respectively. CFA demonstrated reasonable model fit with, SRMR = 0.058, RMSEA = 0.052, CFI = 0.921 and TLI = 0.910. In CFA factor loading value ranged from 0.408 to 0.765. In CFA all the item loaded to their respective scales with a success rate of 100%. Table 2 shows the loading of SGRQ-CM in 3 factor structure model.

**Table 2:** Exploratory factor Loadings for the Malaysian version of St George Respiratory COPD specific questionnaire (SGRQ-CM)

	**Explanatory Factor Analysis (EFA)**			**Confirmatory Factor Analysis (CFA)**		
Variables	Activity	Symptoms	Impact	Activity	Symptoms	Impact
**Q1:** I cough		0.536			0.663	
**Q2:** Phlegm was expelled when I cough	0.414	0.577			0.712	
**Q3:** I have shortness of breath	0.376	0.473			0.538	
**Q4:** I have attacks of wheezing		0.450			0.604	
**Q5:** How many attacks of chest problem did you have during the last year?		0.640			0.728	
**Q6:** How often do you have good days (with not many chest problems)?		0.517	0.470		0.571	
**Q7:** If you experience wheezing, is it worse in the morning?			−0.510		0.412	
**Q9.1:** Make you feel breathless getting washed or dressed	0.700			0.839		
**Q9.2:** Make you feel breathless walking around the home	0.473			0.555		
**Q9.3:** Make you feel breathless walking outside on flat ground	0.329			0.416		
**Q9.4:** Make you feel breathless walking up a flight of stairs		0.540		0.424		
**Q9.5:** Make you feel breathless walking up hills	0.568	0.383		0.642		
**Q12.1:** I take a long time to take a bath or put on clothes	0.284			0.410		
**Q12.2:** I cannot take a bath or shower, or I take a long time	0.371			0.524		
**Q12.3:** I walk slower than other people, or I stop for rests	0.488	0.443		0.519		
**Q12.4:** Jobs such as housework take a long time, or I have to stop for rests	0.621			0.765		
**Q12.5:** If I walk up one flight of stairs, I have to go slowly or stop	0.517			0.561		
**Q12.6:** If I hurry or walk fast, I have to stop or slow down	0.352			0.434		
**Q12.7:** My breathing makes it difficult to do things such as walk up hills, carrying things upstairs, light gardening such as weeding, dance, play bowling or play golf	__	__	__	__	__	__
**Q12.8:** My breathing makes it difficult to do things such as carry heavy stuff, dig the garden or gardening, jog or walk at 5 miles per hour, play tennis or swim	0.487			0.559		
**Q8:** How would you describe your chest condition?		0.477				0.398
**Q10.1:** My cough hurts			0.534			0.568
**Q10.2:** My cough makes me tired			0.516			0.587
**Q10.3:** I am breathless when I talk			0.384			0.454
**Q10.4:** I am breathless when I bend over			0.270			0.416
**Q10.5:** My cough or breathing disturbs my sleep			0.451			0.632
**Q10.6:** I get exhausted easily		0.375	0.510			0.563
**Q11.1:** My cough or breathing is embarrassing in public			0.514			0.538
**Q11.2:** My chest problem is a nuisance to my family, friends or neighbors			0.302			0.464
**Q11.3:** I get scared or panic when I cannot breathe			0.289			0.408
**Q11.4:** I feel that I am not in control of my chest problem		0.533				0.434
**Q11.5:** I have become weak or an invalid due to my chest problems	0.364		0.565			0.731
**Q11.6:** Exercise is not safe for me			0.445			0.508
**Q11.7:** Everything seems too much of an effort			0.615			0.663
**Q13.1:** I cannot play sports or games			0.380			0.512
**Q13.2:** I cannot go out for entertainment or recreation			0.343			0.459
**Q13.3:** I cannot go out of the house to do the shopping		0.486				0.429
**Q13.4:** I cannot do housework			0.497			0.624
**Q13.5:** I cannot move far from my bed or chair			0.360			0.439
**Q14:** How does your chest problem affect you?		−0.659	−0.299			0.568

^__^Removed item due to factor cross loading

Scaling properties, internal consistency and test-retest reliability results are presented in Table 3. Approximately 2.1% of patients showed the best possible score in symptom components and 4.3% of patients showed the best possible score in activity component. None of the patients showed worst possible score. SGRQ-CM and three subscales showed excellent internal consistency. The Cronbach alpha coefficient for SGRQ-CM total score, symptom subscale, activity subscale and impact subscale was 0.87, 0.74, 0.70 and 0.71 respectively. No significant difference was observed for SGRQ-CM total and subscale scores at baseline and at 2 weeks visit (*p* > 0.05). At two weeks interval, ICC for total SGRQ-CM, symptom subscale, activity subscale and impact subscale was reported as 0.88, 0.89, 0.85 and 0.90 respectively.

**Table 3:** Results from tests of scaling properties of Malaysian translated version of the St George’s Respiratory COPD specific Questionnaire

	**Symptom**	**Activity**	**Impact**	**SGRQ-CM**
Items n	7	13	20	40
Theoretical Range	0–100	0–100	0–100	0–100
Observed range	8.88–100	22.39–100	9.23–90.61	17.59–92.32
At ceiling % (n) ^a^	2.1 (3)	4.3 (6)	0	0
At Floor % ^b^	0	0	0	0
Item Convergent Validity ^c^	0.44–0.79	0.14–0.72	0.13–0.68	__
Scaling Success % ^d^	87.5	84.6	80	__
Cronbach’s α Coefficient	0.74	0.70	0.71	0.87
Test-Retest reliability ^e^				
Mean Difference ^f^	−3.02766	−.78737	−1.37505	−1.44755
ICC	0.89	0.85	0.90	0.88

^a^ Best possible score; ^b^ Worst possible score; ^c^ range of correlation between item and their hypothesized scale; ^d^ Percentage of items correlated higher with the hypothesized subscale than other subscale; ^e^ test retest was performed on 48 patients; ^f^ Mean difference in baseline and 2 weeks SGRQ-CM scores; *n* number, *ICC* Intraclass correlation coefficient, *SGRQ-CM* St George’s Respiratory COPD specific Questionnaire Malaysian version

As shown in Table 4, SGRQ-CM showed good correlation with the CAT, EQ-5D-5 L UI, EQ-VAS and mMRC dyspnea scale and low but significant correlation with FEV_1_%predicted in the expected direction. Correlation of SGRQ-CM with CAT, EQ-5D-5 L UI, EQ-VAS, mMRC dyspnea scale and FEV_1_% predicted was reported as 0.86, − 0.82, − 0.61, 0.72 and − 0.42 respectively. Correlation coefficient among the subscales and other clinical and health status measures ranged from r = − 0.31 to r = − 0.87.

**Table 4:** Results of correlation between baseline SGRQ-CM scores and other clinical and health status measures

	**Symptom**	**Activity**	**Impact**	**Total**
FEV_1_/FVC	−0.35	−0.46	−0.42	−0.46
FEV_1_%predicted	−0.31	− 0.42	− 0.40	− 0.42
CAT	0.79	0.76	0.87	0.86
EQ-5D-5 L UI	−0.71	− 0.74	− 0.80	−0.82
EQ-VAS	−0.53	−0.59	− 0.56	−0.61
mMRC Dyspnea	0.64	0.79	0.63	0.72

Results from Pearson correlation Coefficient test; Correlation is significant at the 0.05 level (2-tailed); High % Predicted FEV1 and EQ-5D-5 L score indicate better quality of life; CAT COPD assessment test; EQ-5D-5 L UI, European Quality of life 5-Dimension 5-Level questionnaire Utility index score; EQ-VAS, European Quality of life 5-Dimension 5-Level questionnaire Visual analogue score; FEV_1_, forced expiratory volume in one second; FVC, Forced vital capacity; FEV_1_ (%), percentage predicted FEV_1_; mMRC modified medical research council dyspnea scale; spirometric data were post-bronchodilator

Responsiveness of the SGRQ-CM to detect changes in health status over time was presented in Table 5. Patients were followed up for six months. Among 240 included patients twenty seven patients lost the follow-up due to different reasons (changed hospital, moved from the city and surgery). 213 patients completed the follow up visits. The mean difference in % predicted FEV_1_ values between baseline and 6 months follow-up was insignificant (*p* = 0.13), whereas, a significant difference was observed in the mean differences of SGRQ-CM total and subscale scores between the two occasions (*p* < 0.001).

**Table 5:** Mean difference in clinical and health status measures at baseline and after 6 months of follow-up (*n* = 213)

	Mean Difference (95% CI)	Effect Size	*p* value
**SGRQ-CM**			
Symptom	−2.29 (−3.14–1.43)	0.38	< 0.001
Activity	−1.51 (−2.52–0.48)	0.27	< 0.01
Impact	−2.03 (−3.0–1.06)	0.35	< 0.001
Total	−1.73 (−2.62–0.84)	0.30	< 0.001
**Spirometry Values**			
FEV_1_/FVC	0.54 (−0.09–1.18)	0.09	0.09^a^
FEV_1_ (%)	0.57 (−0.17–1.31)	0.10	0.13^a^

^a^ Mean difference is non-significant; FEV_1_, forced expiratory volume in one second; FVC, Forced vital capacity; FEV_1_ (%), percentage predicted FEV_1_; Spirometric data were post-bronchodilator; SGRQ-CM, Malaysian version of St George’s Respiratory COPD specific Questionnaire

Significant mean difference (95% CI) was observed in total and component scores of the patients at different levels of disease severity [GOLD IV vs III, 10.78 (1.38–20.17), *p* = 0.02; and GOLD III vs II, 8.26 (1.27–15.26), *p* < 0.01]. The mean difference (95% CI) in SGRQ-CM scores in patients at mMRC dyspnea grade IV vs III was 8.59 (2.59–14.59), p < 0.01 (Table 6). MCID using anchor-based approach was reported as the mean difference in SGRQ-CM impact subscale score between dyspnea grade IV vs III patients, as it differentiates the house bound patients from patients who can perform their normal activities. Using anchor-based approach the MCID was calculated as 5.07 (95% CI -2.54-12.67).

**Table 6:** Mean difference in SGRQ-CM scores between patients at different levels of disease severity

	**Activity Subscale**			**Impact Subscale**			**SGRQ-CM Total Score**		
	Mean Difference (95% CI)	Effect Size	*p value*	Mean Difference (95% CI)	Effect Size	*p* value	Mean Difference (95% CI)	Effect Size	*p* value
*GOLD Classification*									
IV vs III	9.35 (0.05–18.64)	0.34	0.04	12.04 (1.45–22.63)	0.45	0.02	10.78 (1.38–20.17)	0.37	0.02
III vs II	9.51 (2.58–16.43)	0.33	< 0.01	8.91 (1.02–16.79)	0.31	0.00	8.26 (1.27–15.26)	0.28	< 0.01
mMRC Dyspnea grade									
IV vs III	14.58 (9.33–19.83)	0.58	< 0.001	5.07 (2.54–12.67)	0.37	0.19	8.59 (2.59–14.59)	0.43	< 0.01
III vs II	8.89 (3.39–14.39)	0.39	< 0.01	10.57 (2.60–18.54)	0.49	0.01	9.86 (3.57–16.15)	0.46	< 0.01

GOLD Global Initiative for Obstructive Lungs disease; GOLD II, FEV1 50 to 80% predicted; GOLD III, FEV1 30 to 50% predicted; GOLD IV, FEV1 < 30% predicted; mMRC modified medical research council dyspnea scale; SGRQ-CM, Malaysian version of St George’s Respiratory COPD specific Questionnaire

Mean difference (95% CI) in SGRQ-CM impact and activity subscale scores during hospital admission and after one month of discharge from hospital were displayed in Table 7. Using distribution-based approach MCID was calculated as 6.05 (5.30–6.80).

**Table 7:** Mean difference in clinical and health status measures during exacerbation and after one month of treatment (*n* = 47)

	**Mean Difference (95% CI)**	**Effect Size**	***p*value**
SGRQ-CM			
Activity	5.94 (5.29–6.58)	0.47	< 0.001
Impact	6.05 (5.30–6.80)	0.52	< 0.001
Spirometry Values			
FEV1/FVC	−1.57 (−1.85–1.30)	0.18	< 0.001
FEV1 (%)	−1.58 (−1.86–1.31)	0.19	< 0.001

Exacerbation Patients n = 47; difference is significant at 0.05 level; FEV_1_, forced expiratory volume in one second; FVC, Forced vital capacity; FEV_1_ (%), percentage predicted FEV_1_; Spirometric data were post-bronchodilator; SGRQ-CM, Malaysian version of St George’s Respiratory COPD specific Questionnaire

## Discussion

Our results demonstrated highly acceptable reliability, validity and responsiveness of SGRQ-CM in Malaysian COPD patients. The results are in line with the results of original SGRQ-C results and suggests that the Malaysian version of SGRQ-C is conceptually equal to original SGRQ-C and is considered valid to use in Malaysian population. The SGRQ-CM questionnaire and all the three subscales showed good to excellent internal consistency and test retest reliability. As reported in literature, internal consistency is considered good if Cronbach’s α is greater than 0.7 [25]. The Cronbach’s α coefficient for total and all the subscales was reported above 0.7. The Cronbach’s α coefficient values were comparable to published data. It was slightly greater than that reported in similar validation study of Chinese version of SGRQ-C [35]. The Cronbach’s α coefficient for Chinese version of SGRQ-C was reported from 0.59 to 0.95 [35]. Cronbach’s alpha was slightly lesser than other similar validation study of the Spanish and the Persian versions of SGRQ-C [36, 37]. In the Spanish version of SGRQ-C cronbach’s alpha was reported as (0.72 to 0.94) and in Persian version it was reported as (0.699 to 0.916) [36, 37]. The small difference in the alpha value shows the possibility of the strong impact of culture on activity and impact subscale.

The SGRQ-CM showed significant correlation with other health status measures in this study. The total SGRQ-CM score showed high correlation, while all subscales showed good correlation with CAT, EQ-5D-5 L and mMRC. Similarly, to the original version of SGRQ-C, the activity subscale showed highest correlation with dyspnea scale and impact subscale showed highest correlation with other health status measure [5]. The correlation of SGRQ-C total and subscale scores was stronger with EQ-5D-5 L UI as compared to EQ-VAS. The SGRQ-CM and all subscales showed higher correlation with CAT as compared to the EQ-5D-5 L and mMRC. The highest correlation in impact subscale and CAT shows the specificity of SGRQ-CM to measure the activity limitation specifically due to the COPD [38]. A negative correlation was observed between SGRQ-CM scores and FEV_1_% predicted. Patients with higher predicted FEV_1_ scores reported better QoL as compared to patients with a low predicted FEV_1_ score. This shows SGRQ-CM can accurately distinguish patients at different disease stages. A significant but low correlation between SGRQ-CM and predicted FEV_1_ was as per expectations. In COPD patients, literature showed a weak relationship between spirometric values and the original SGRQ-C scores. However, the correlation was comparably higher with SGRQ-CM scores than reported in the previous studies [7, 9]. Activity component showed highest correlation with predicted FEV_1_. Low correlation between SGRQ-CM and FEV_1_% predicted, than between SGRQ-CM and other health status measures gives a better picture of disease severity under the influence of different factors as compared to clinical symptoms.

A health status measure should be able to discriminate patients, according to severity of disease and evaluate the difference in the QoL over time [39]. The effect size gives the estimate of the power of an instrument to differentiate between patients in different stages of disease severity and to evaluate the impact of an intervention. Despite no significant difference in spirometric values at baseline and 6 months, a significant difference was observed in SGRQ-CM total and subscale scores at baseline and 6 months. These results endorse the previous research, that small deviation in lung function considerably affects the QoL of COPD patients [40]. This shows the sensitivity of SGRQ-CM in detecting the health status change as compared to clinical measures. The ability of a health status measure to detect change in health status in COPD patients is important as, health status is the predictor of mortality and COPD exacerbation in COPD patients [41, 42].

In consistent with the original version of SGRQ-C, the Malaysian version was found to be optimal with three factor structure. In EFA maximum items showed loading in their relevant subscales except few items i.e. the item 7 (If you have a wheeze, is it worse in the morning?) is more related to the symptoms but it showed loading in impact subscale. Similarly item 9.4 showed loading in symptom factor instead of activity factor and item 8, 11.4, 13.3 and 14 showed loading in symptom subscale instead of impact subscale. But in CFA all the items showed loading in their relevant subscales consistent with the previous research. Moreover, factor loadings value > 0.4 were observed for majority of the items, demonstrating the significant contribution of these items in assessment of health status.

MCID is the minimal difference in outcome measure that clinicians and patients would care about. According to Jaeschke and colleagues a strong MCID should be reflective of patient reported outcomes and clinical findings [43]. Both Anchor-based approach and distribution-based approach were used to calculate MCID which represents patient reported outcomes and clinical findings respectively. Using anchor-based approach and distribution-based approach the MCID was calculated as 5.07 (95% CI -2.54-12.67) and 6.05 (5.30–6.80) respectively. The MCID reported in our study is comparable to the one reported in literature. In Chinese version of SGRQ-C the MCID was reported as 6.6 (95%CI – 0.8–14.1) [7]. MCID calculated by all the parameters was greater than recommended MCID of 4 units calculated for original SGRQ-C [31]. The difference in our reported values and the original SGRQ-C may be due to difference in study population and difference in cultural values of Asian and European population. There is no universally accepted method for calculation of MCID. Utilization of maximum possible perspectives to calculate MCID will enhance the interpretability of the instrument. Moreover, a significant difference in SGRQ-C scores in patients suffering from exacerbation and after one month of discharge from hospital shows that SGRQ-C can reliably identify response to therapy in COPD patients.

Until now, generic QOL questionnaires have been used in clinical trials and other research studies in Malaysian COPD patients. Generic questionnaires are considered as less sensitive to changes in health status, as compared to disease specific questionnaires [17, 44]. This limits their use in clinical trials. To the best of authors’ knowledge SGRQ-CM is the first COPD specific QoL instrument to be validated for use in COPD patients in Malaysia. The present study has several strengths. The quality of the study was assessed using COSMIN checklist. Among the ten measurement properties mentioned in COSMIN checklist to assess the good methodological quality of a validation study, nine (internal consistency, reliability, content validity, structural validity, hypotheses testing, cross-cultural validity, criterion validity, responsiveness and interpretability) were reported according to the set standards [45]. The sample size may not be large enough as compared to number of items in the scale to perform psychometric validation, but the sample size used for the current study was higher than most of the previously published research on validation of SGRQ-C [6–9]. Longitudinal design allowed more detailed investigation of SGRQ-C correlation with changing health status over the period of time, including exacerbations. Moreover, utilization of anchor-based approach and distribution-based approach to calculate MCID strengthens the results of current research. Despite several strengths few limitations should be kept in mind before interpreting the results. Although larger sample size was used than previous studies, the sample size was still limited for test-retest reliability. Usually one fifth of the total sample size is recommended for the test-retest reliability. The number of patients included in “test-retest reliability” test was according to the above-mentioned recommendation. The mean age reported in the study is bit higher, but it is in line with the previously published research on COPD in Malaysia [46]. The study was single centered, so the results should be generalized with caution. However, the study population is likely to be representative of general population as Penang Hospital is one of the leading Hospitals of South Malaysia and provides services to the majority of population of South Malaysia.

## Conclusion

In conclusion SGRQ-CM has a strong evidence of validity (construct and concurrent), reliability and responsiveness to disease severity in Malaysian COPD patients. SGRQ-C is a reliable instrument to be used for assessment of overall health status of COPD patients and long term follow-up. It can identify the key areas of health problem that health care professional can explore further during consultation. It can also be used as a reliable QoL measure in future research, including randomized clinical trials, rehabilitation studies, and QoL studies in COPD patients. MCID and responsiveness test results shows that it can reliably assess the impact of an intervention in COPD patients and can be used in clinical practice to measure treatment efficacy for selection of optimal treatment.

## Data Availability

Data is available on request from corresponding author.

## Abbreviations

GOLD: Global Initiative for Chronic Obstructive Lung Disease
COPD: Chronic obstructive lung disease
QoL: Quality of life
MCID: Minimum clinically important difference
FEV_1_: Forced expiratory volume in one second
FVC: Forced vital capacity
FDA: Food and Drug Administration Authority
mMRC: Modified Medical Research Council dyspnea scale
CAT: COPD assessment test
EQ-5D-5 L UI: European quality of life 5-dimension 5-level questionnaire Utility index score
EQ-VAS: European Quality of life 5-dimension 5-level questionnaire Visual analogue score
SGRQ-C: St George’s respiratory COPD specific questionnaire
SGRQ-CM: St George’s respiratory COPD specific questionnaire Malaysian version
EFA: Explanatory factor analysis
CFA: Confirmatory factor analysis
ICC: Intraclass correlation coefficients
